# Research Trends and Hotspots of Extracorporeal Membrane Oxygenation: A 10-Year Bibliometric Study and Visualization Analysis

**DOI:** 10.3389/fmed.2021.752956

**Published:** 2021-10-26

**Authors:** Huaqin Wang, Siqi Deng, Xiaoyu Fan, Jinxiu Li, Li Tang, Yamin Li, Bo Yu

**Affiliations:** ^1^Clinical Nursing Teaching and Research Section, The Second Xiangya Hospital of Central South University, Changsha, China; ^2^College of Nursing, Hunan University of Traditional Chinese Medicine, Changsha, China; ^3^College of Medicine, Jishou University, Jishou, China; ^4^Department of Critical Care Medicine, The Second Xiangya Hospital of Central South University, Changsha, China

**Keywords:** ECMO, visualization analysis, bibliometric study, research trends and hotspots, COVID-19

## Abstract

**Objective:** To determine the research hotspots and trends in the field of extracorporeal membrane oxygenation (ECMO), and to provide a reference for further and wider research in the future.

**Methods:** The literatures on ECMO from January 2011 to July 2021 in the Web of Science Core Collection (WOSCC) database were searched, and Citespace5.8.R1 software was used to conduct bibliographic and visual analysis on the literature by country, institution, author and keywords.

**Results:** A total of 5,986 articles were enrolled. According to an observation, the number of articles published in the past decade has increased, especially from 2019 to 2020. The USA had the largest number of publications, while less ECMO related studies were conducted among non-developed countries. The University of Michigan (Univ Michigan) was the institution that had the largest number of publications and the highest centrality, and Daniel B was the author who had the largest number of publications. However, more inter-institutional cooperation among author teams was needed. The focus of existing ECMO research has primarily been on the treatment of patients suffering from severe cardiopulmonary failure, and the prevention and management of complications during the application ECMO.

**Conclusion:** Inter-regional and inter-institutional cooperation and exchanges should be carried out among ECMO research teams and institutions. The suggested research direction is to further broaden the application scope of ECMO, while determining the ways to reduce the incidence of complications and the cost, cultivate specialized team talents, and promote the application thereof.

## Introduction

Extracorporeal membrane oxygenation (ECMO), an artificial *in vitro* support system, is commonly used to treat refractory heart and respiratory failure (1, 2). As a form of extracorporeal life support system, ECMO can support the respiratory function of patients with respiratory failure, so as to alleviate circulatory hypoxemia in patients developing cardiopulmonary failure. Therefore, the function of ECMO is not limited to extracorporeal circulation support. ECMO has been extensively applied in clinical cardiac surgery, respiratory diseases and critical care medicine for the past 50 years. In particular, the outbreak of Coronavirus disease 2019 (COVID-19) in late 2019 has posed a significant threat to human health and a huge challenge for global public health security (3, 4). ECMO plays an important role in saving lives as a rescue therapy for COVID-19 patients (5–7). Due to the precise therapeutic effects thereof, ECMO technology has become mature in clinical practices rapidly, and a large number of studies have been performed at the same time. Bibliometrics, an interdisciplinary science, involves mathematical and statistical tools to identify trends, as well as research themes or areas of focus (8, 9). In bibliometrics, based on multiple indicators such as references, authors, journals, countries and institutions, visualizations are generated, which provides an in-depth assessment of thematic trends and priorities in a given field (10–12). Most previous studies on ECMO have centered on theoretical research and experience sharing. Meanwhile, quantitative and visual analysis methods have not been adopted to explore the vertical and horizontal characteristics, development and multiple impacts of the present topic. As such, in the present study, the bibliometric analysis software (CiteSpace) was used to conduct statistics and analysis of ECMO related literatures in the past 10 years, and to generate visual graphics for the exploration of the hot spots and future development trends in the present research field, so as to facilitate further research in the future.

## Methods

### Data Acquisition and Search Strategy

Web of Science Core Collection (WOSCC) was used as the source for retrieval and screening, with literatures on ECMO from January 2011 to July 2021 being retrieved. The key words included “Extracorporeal Membrane Oxygenation” and “ECMO.” The inclusion criteria included literatures with “ECMO” as the main research content. The exclusion criteria are as follows: newspapers; advertisements; scientific and technological achievements; books and conference papers; repeated publications; and literature with incomplete information. The subjects and abstracts were read independently in pairs and screened on the basis of inclusion and exclusion criteria. If the title and abstract of a study could not be determined, the full text was read, and then a third researcher would be consulted to help decide whether such study should be included or not.

### Analysis Software

**Figure 1:**
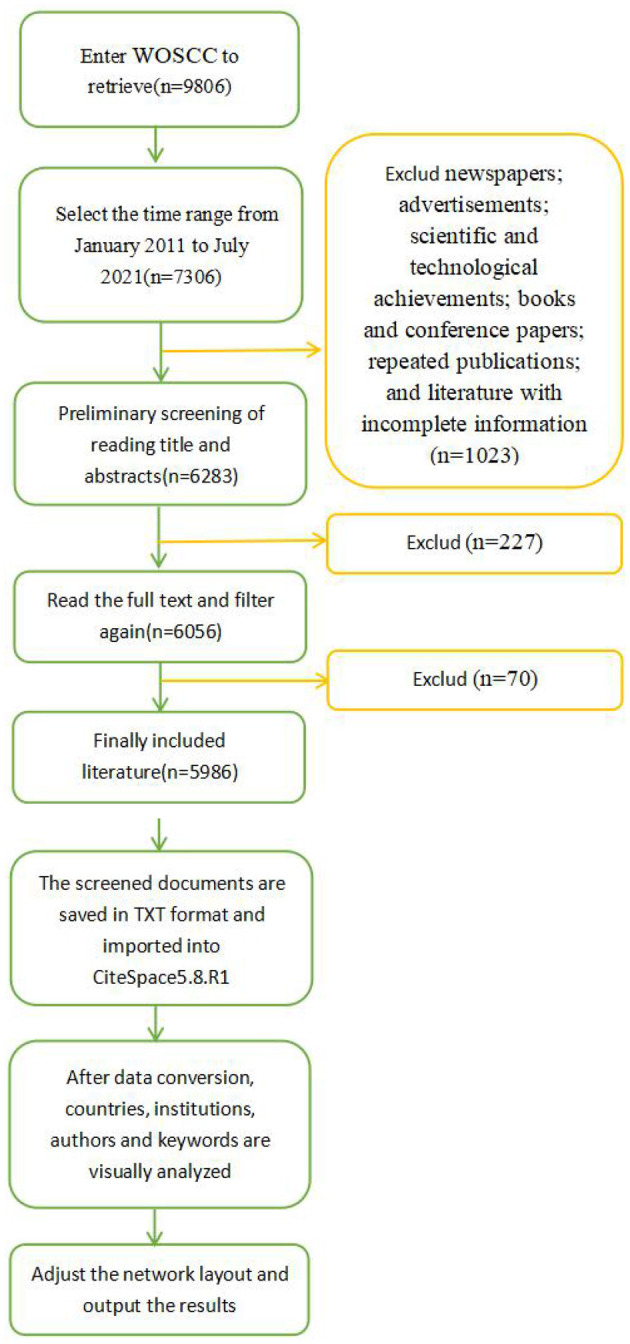
Visual analysis flow chart of ECMO research field.

The included literatures were exported in TEXT format, and then imported into Citespace5.8.R1 software that was used for data visualization analysis and bibliometric analysis. The overall visual analysis process was shown in Figure 1. The span of literature in the present study was selected as 1 year. By adjusting corresponding parameters, co-occurrence analysis, cluster analysis and visualization map were performed for countries, authors, institutions and keywords. Frequency was applied to represent the number of countries, institutions and authors. To measure the importance of nodes in the network, the centrality was used, with a higher centrality representing a higher degree of importance. Different nodes in the visualization represent different countries/regions, institutions, authors, or keywords. The size of the node marks the frequency or centrality of the literature. A line between nodes refers to a cooperative network.

### Statistical Methods

Frequency, the main metric, was used to identify the core countries/territories, institutions, authors, and keywords. Centrality means betweenness centrality that is an indicator to measure the importance of nodes in the network. This indicator was used by Citespace to discover and measure the significance of various kinds of literatures and purple circles were utilized to highlight such literatures. Pieces of literatures with high betweenness centrality were usually the key hubs connecting two different fields. It is also called a turning point in Citespace. This method of calculating the importance of nodes was proposed by Freeman in 1977. Betweenness centrality is calculated as follows:


(1) $$\begin{array}{r} BC_{\text{i}}=\underset{\text{s}\neq i\neq t}{\sum }\frac{n_{st}^{i}}{g_{st}} \end{array}$$

In the formula, *g*_*st*_ is the number of shortest paths from node *s* to node *t*, and means the number of shortest paths through node i among *g*_*st*_ shortest paths from node *s* to node t. From the perspective of information transmission, the higher the betweenness centrality is, the greater the importance of the node is. The result of clustering analysis is a keyword co-occurrence network. The cluster view emerges the distribution of fields from a different point of view. The timeline view primarily reveals solicitude to delineate the relationship between clustering results and concentrates on the historical span of literatures in a clustering result.

## Results

A total of 9,806 studies were obtained through preliminary retrieval, and 5,986 studies were included after screening according to the inclusion and exclusion criteria.

### Annual Publication Trend of Literature

**Figure 2:**
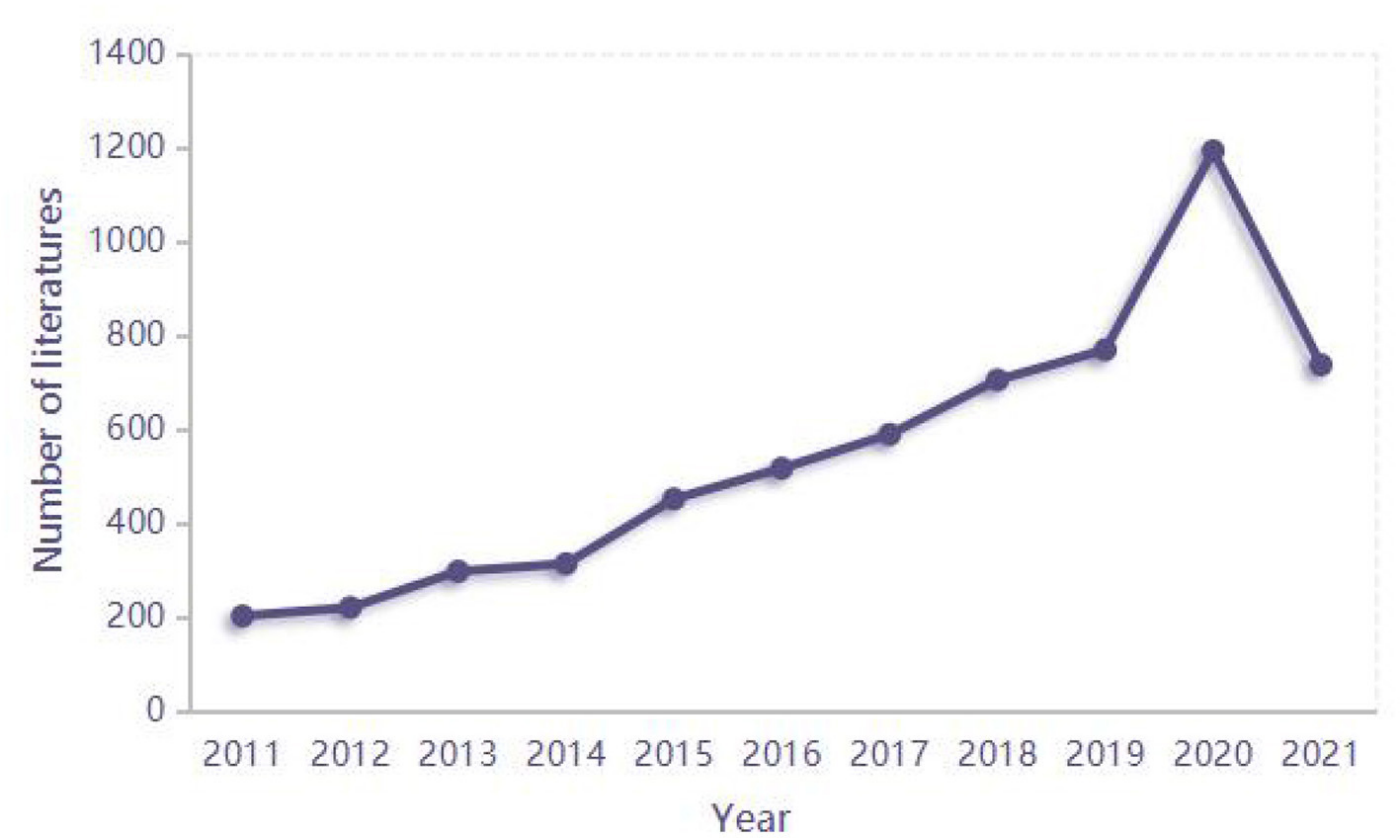
Annual publications.

In the past 10 years, the number of published studies on ECMO exhibited an overall upward trend, especially during the period from 2019 to 2020 (Table 1; Figure 2). The number of literatures published in 2020 was five times that of 2011 due to the outbreak of COVID-19 in late 2019. ECMO has obtained worldwide attention as an effective treatment, and extensive studies have been conducted on the indications and efficacy thereof.

**Table 1:** Literature annual distribution.

**Year of publication**	**Record**	**% of 5,986**
2021	736	12.30
2020	1,191	19.90
2019	768	12.83
2018	704	11.76
2017	588	9.83
2016	516	8.62
2015	451	7.53
2014	313	5.23
2013	297	4.96
2012	220	3.68
2011	202	3.37

### State Issuance of Documents

**Figure 3:**
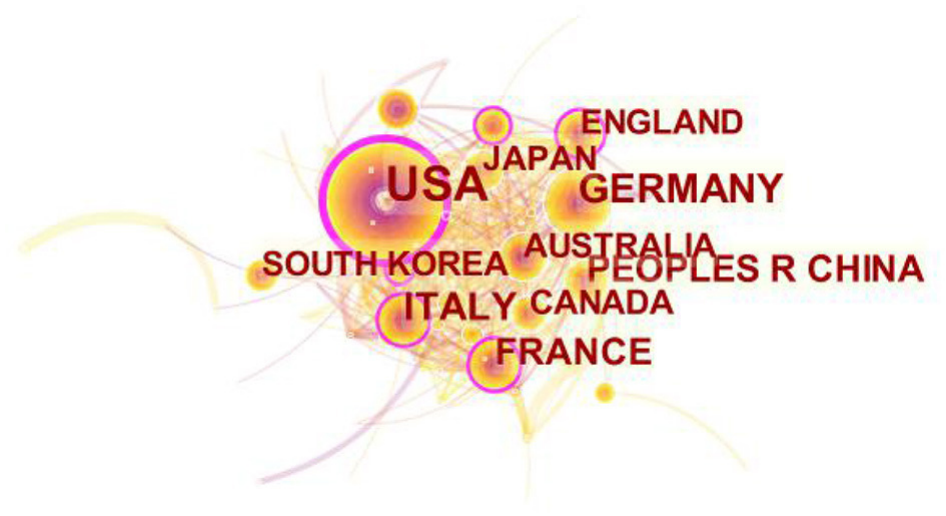
Country visualization map.

According to the frequency of publication and centrality, the countries that published the included literatures were analyzed and ranked, respectively. The top 10 countries were listed in Table 2, in which the USA ranked the first in the frequency of publication and centrality. Each node in the country visualization map represents a country, the size of the node stands for the amount of output, and the lines between the nodes are the partnerships between countries (Figure 3).

**Table 2:** Top 10 countries by publication frequency and centrality.

**No**.	**Country**	**Frequency**	**Country**	**Centrality**
1	USA	1,882	USA	0.22
2	Germany	646	France	0.16
3	Italy	384	Italy	0.14
4	China	313	England	0.12
5	France	304	Germany	0.10
6	England	241	Japan	0.07
7	South Korea	234	Australia	0.05
8	Japan	233	Canada	0.04
9	Australia	223	China	0.01
10	Canada	188	South Korea	0.00

### Institutional Publication Status

**Figure 4:**
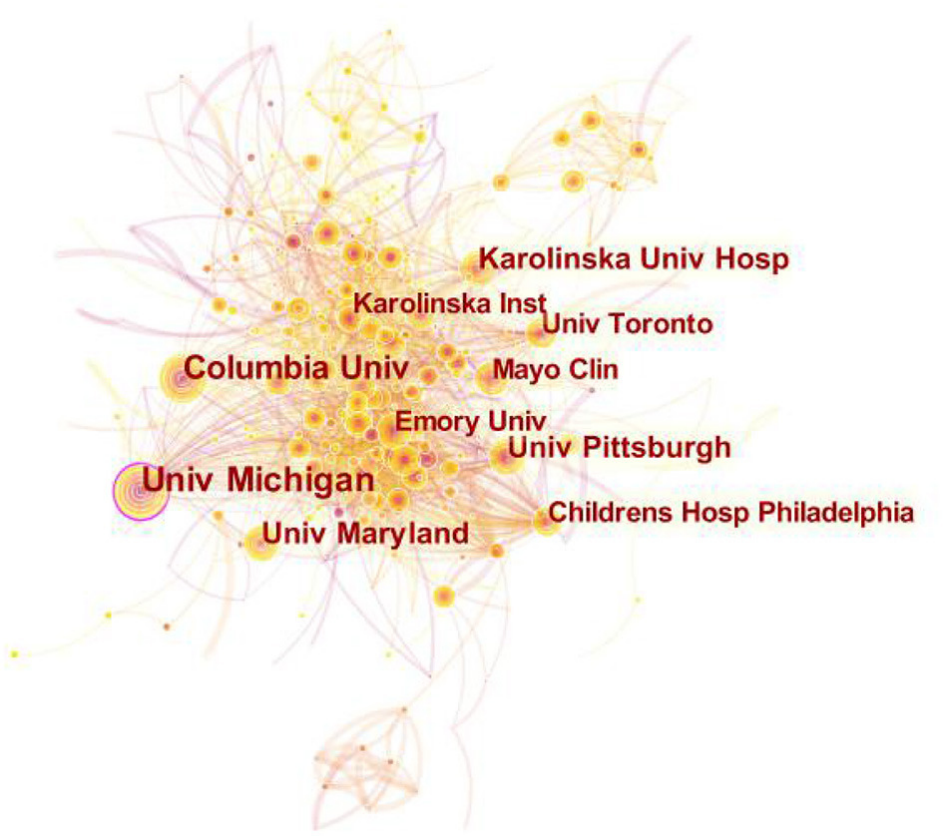
Institution visualization map.

Research institutions were ranked by publication frequency and centrality, and an institution visualization map was created (Figure 4). The top 10 institutions are listed in Table 3, in which the University of Michigan (Univ Michigan) ranked first with 144 papers, and also ranked first with 0.13 centrality.

**Table 3:** Top 10 institutions by publication frequency and centrality.

**No**.	**Institution**	**Frequency**	**Institution**	**Centrality**
1	Univ Michigan	144	Univ Michigan	0.13
2	Columbia Univ	124	Univ Toronto	0.08
3	Univ Maryland	81	Childrens Hosp Philadelphia	0.07
4	Karolinska Univ Hosp	75	Univ Pittsburgh	0.06
5	Univ Pittsburgh	74	Columbia Univ	0.04
6	Univ Toronto	73	Emory Univ	0.04
7	Emory Univ	68	Karolinska Inst	0.03
8	Karolinska Inst	67	Karolinska Univ Hosp	0.02
9	Childrens Hosp Philadelphia	64	Mayo Clin	0.02
10	Mayo Clin	63	Univ Maryland	0.01

### Author Publication Status

**Figure 5:**
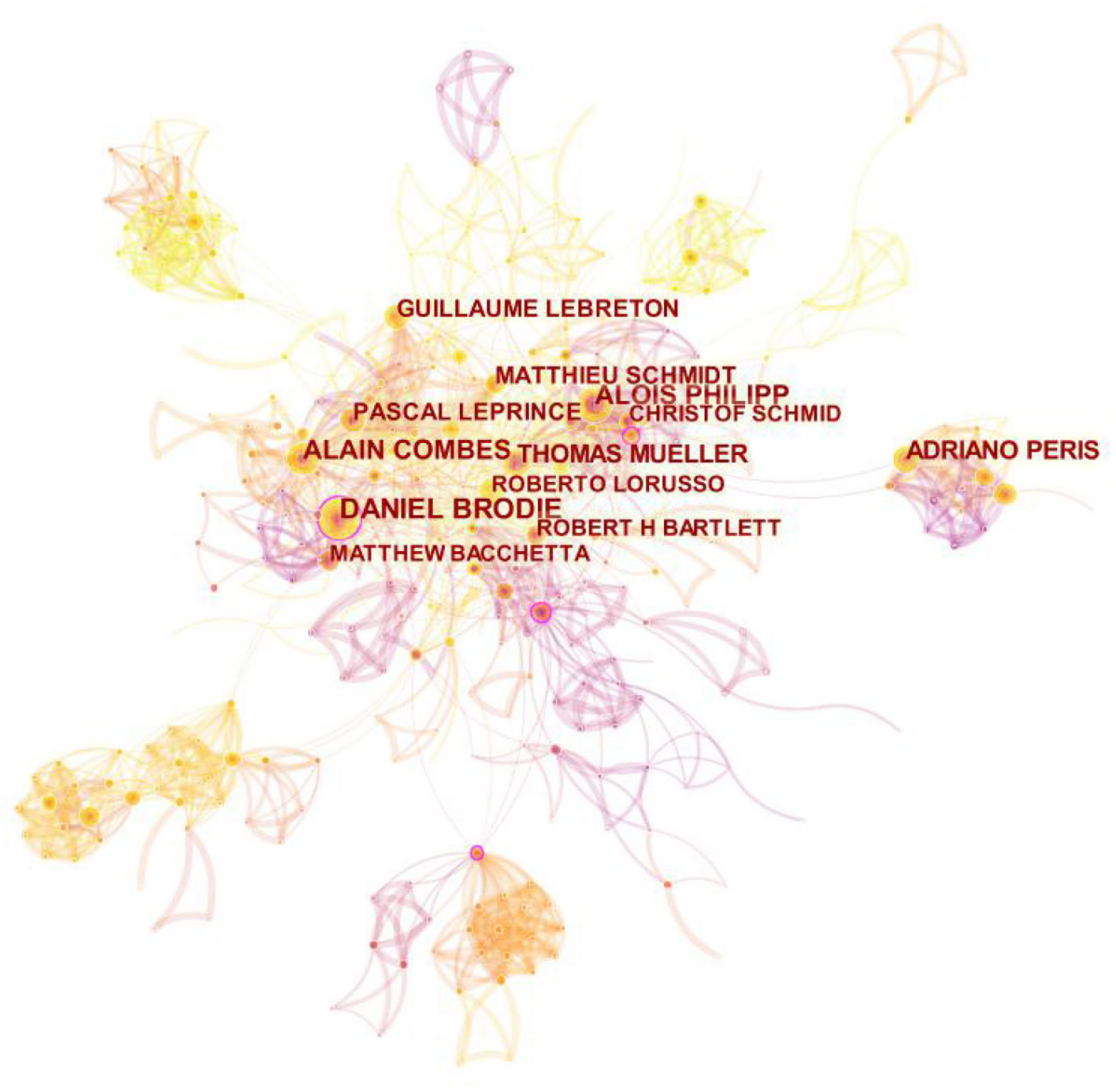
Visualization map of authors.

The authors were ranked by publication frequency and centrality, and an author visualization map was created (Figure 5). The top 10 authors are shown in Table 4, in which the results reveal that Daniel B ranked first with a publication frequency of 73 and a centrality of 0.13.

**Table 4:** Top 10 authors by publication frequency and centrality.

**No**.	**Author**	**Frequency**	**Author**	**Centrality**
1	Daniel B	73	Daniel B	0.13
2	Alain C	60	Alain C	0.08
3	Alois P	50	Roberto HB	0.08
4	Thomas M	45	Thomas M	0.06
5	Adriano P	41	Alois P	0.04
6	Guillaumel L	37	Adriano P	0.03
7	Matthieu S	36	Matthieu S	0.03
8	Pascal L	36	Guillaumel L	0.01
9	Matthew B	33	Matthew B	0.01
10	Roberto HB	33	Pascal L	0.00

### Keywords Clustering Timeline and Keyword Bursts

**Figure 6:**
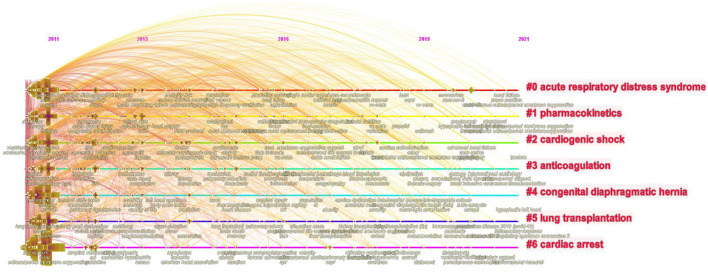
Timeline view.

**Figure 7:**
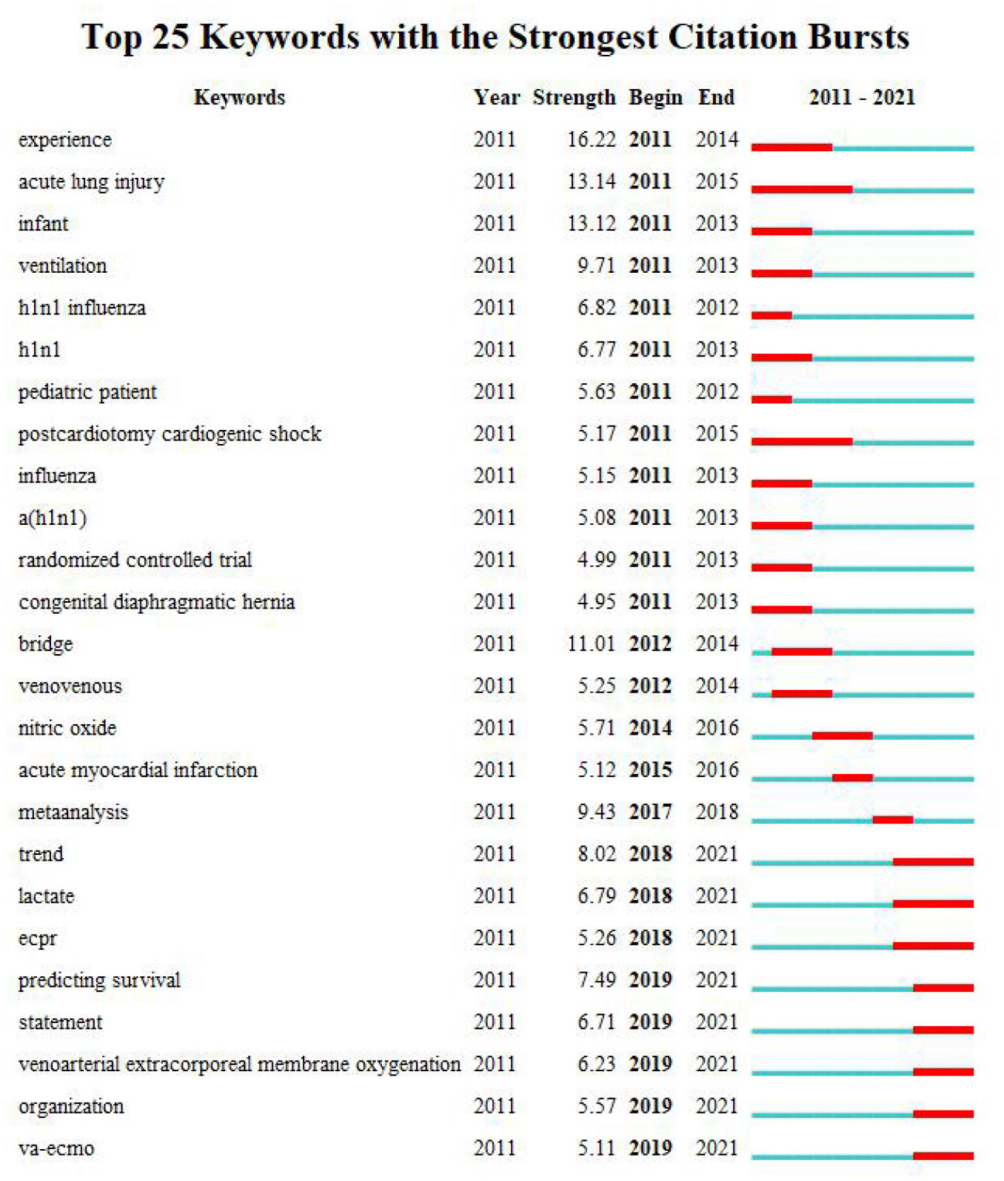
Burst test of keywords.

Cluster analysis was formed on the basis of keyword co-occurrence, and seven clusters were formed in total: acute respiratory distress syndrome, pharmacokinetics, cardiogenic shock, anticoagulation, congenital diaphragmatic hemia, lung transplantation, and cardiac arrest. TimeLine View was used to draw a timeline for keywords after clustering, and the length of the horizontal line corresponding to each cluster represents the time span of the cluster, as shown in Figure 6. TimeLine View mainly reveals the relationship between description clustering results, and focuses on the historical span of literature in the clustering results. CiteSpace was further used to detect bursts of keywords with high frequency, as shown in Figure 7.

## Discussion

### Analysis of Countries

In the present study, the top 10 countries in ECMO related fields were found to be mainly developed countries such as the United States, Germany, Italy and others. Both in terms of quantity and quality, developed countries have obvious advantages in research strength. At the same time, the problem of geographical imbalance in research is also reflected. Only three Asian countries, China, Japan and South Korea, were in the top 10 list (Table 2). Studies have shown that despite being effective, ECMO is considerably expensive (13). A study in Turkey also revealed that current ECMO resources are inadequate (14). Hence, ECMO related studies are rarely conducted in non-developed countries, which may be related to the high cost of ECMO. The economic strength of hospitals in non-developed countries is insufficient, and the economic bearing capacity of the majority of patients is also low, so the promotion is difficult to some extent.

### Analysis of Authors and Institutions

The results reveal that many authors collaborated closely with each other and formed several research teams. Among those teams, the top two research teams of Daniel B and Alain C both focused on the treatment and management of acute respiratory distress syndrome (ARDS) with ECMO. From the perspective of the cooperative network of scientific research institutions, Univ Michigan and Columbia University (Columbia Univ) have conducted more research on ECMO, and the centrality of Univ Michigan ranked first. Such results could be attributed to the strong academic atmosphere, strong scientific research foundation and sufficient funds of the two universities. However, the cooperation between institutions is only limited to the cooperation between institutions and the hospitals with which institutions are closely connected, such as The University of Maryland (Univ Maryland) and Children's Hospital of Philadelphia (Childrens Hosp Philadelphia), while the cooperation between different organizations and institutions is less. As such, the cooperation between different institutions needs to be strengthened. The aforementioned results also reflect that researchers have a weak sense of cooperation between different institutions, and further research on ECMO needs to be further explored.

### Research Hotspots Change Greatly Over Time

According to the timeline view (Figure 6) and burst test of keywords (Figure 7), combined with the number of chronological documents, the research on ECMO can be roughly divided into three stages.

The first phase from 2011 to 2013 was the initial exploration of ECMO, and researchers mostly attached attentions on the initial exploration of ECMO's treatment of children suffering from severe cardiopulmonary diseases and patients with influenza A (H1N1) (15–19). At the beginning of the first phase, many scholars found that ECMO was irreplaceable in the treatment of severe respiratory failure in children with severe illnesses, especially newborns and infants (20, 21). However, with the outbreak of H1N1 in 2013, ECMO has become the focus of research, owing to the high fatality rate and the numerous complications such as explosive acute lung injury (ALI) and acute respiratory distress syndrome (9ARDS) (22). ECMO has become a significant factor in reducing patient mortality (23, 24).

In the second phase from 2014 to 2018, ECMO studies exhibited a trend of diversification. ECMO was mainly applied to the treatment of lung transplantation, congenital diaphragmatic hernia and other diseases. For example, Hoetzenecker's (25) team studied the therapeutic effects of ECMO on patients after lung transplantation, while McHoney's (26) team developed the application of ECMO in the treatment of congenital diaphragmatic hernia. At the same time, scholars began to pay attention to various complications that occurred during the application of ECMO (27). The incidence of bleeding and thrombosis as complications is considerably high, and continuous anticoagulation increases the risk of bleeding in patients (28). Some studies have revealed that the incidence of bleeding in adults treated with ECMO is 27–60%. Once severe bleeding events such as intracranial hemorrhage and pulmonary hemorrhage occur, death rate also increases (29). Such complications have significant negative impacts on the quality of patients' life. Determining how to reduce bleeding and prevent the formation of thrombosis has become the top priority in ECMO support therapy (30, 31).

In the third phase from 2019 to 2021, there was an explosion on ECMO, mainly due to the emergence of COVID-19 in late 2019, and the resulting pandemic in 2020. Many researchers have applied previous ECMO experience in treating other diseases to the treatment of COVID-19 patients. As a new life support technology, ECMO not only saved the lives of many patients with COVID-19, but also further improved the success rate of treatment and reduced the incidence of complications. Numerous studies (17, 32, 33) have proved that ECMO is a significant factor in stabilizing and treating survival rates in critically ill COVID-19 patients. Hence, most of the studies in the third phase focused on the exploration and experience of ECMO in the treatment of COVID-19 patients.

### Research Trend and Hotspot Thinking

Understanding the development trend and future trend of a discipline can help us quickly and effectively obtain the latest research hot spots and innovations of relevant disciplines, thus further promoting the efficient development of the discipline. From the salient words and related literatures in recent years, it was obvious that the research focus in this field is single diagnosis and treatment of severe diseases, focusing on the treatment of complications, and gradually deepens into the treatment of multiple diseases. ECMO has been widely used for the treatment of cardiopulmonary diseases that were intractable with conventional treatment for many years. On the other hand, ECMO has played a crucial role in treating severe patients. However, a number of studies (30, 34) have demonstrated that ECMO is accompanied with high complications when treating both mechanically ventilated patients and patients in stable stage. The prevention, diagnosis and treatment of complications are always our focus and difficulty, so we should continue to explore these research hotspots in the future. What's more, there is no unified standard for the timing of ECMO use. For example, Diddle et al. (35) proposed that early use of ECMO before severe arrhythmia, heart failure and other abnormal symptoms in patients with acute myocarditis could improve the prognosis. However, some studies have also suggested that the application of ECMO in patients with organ failure such as low pH, high lactic acid, abnormal liver function, and renal dysfunction requiring renal replacement therapy will lead to the reduce of survival rate (36, 37). According to the current evidence-based medicine, the optimal application time cannot be determined. The precision of ECMO is also the direction of future research.

Most importantly, although the application and development of ECMO have been promoted by the H7N9 avian influenza in 2013 and the COVID-19 epidemic in 2020, the treatment cost of ECMO still remains high, the technology remains complex, and the treatment varies widely among individuals. Further, ECMO exists high risk, and its scope is limited, with some hospitals encountering difficulties in applying such technology. Therefore, how to reduce the cost and improve the portability and mobility will also become the research hotspots and development trends of ECMO. A high-level ECMO team is critical for the improvement of patient's benefit rate. Future research should also pay more attention on the training and establishment of ECMO specialist teams. In the present study, only a single database, namely WOSCC, was retrieved, and other databases were not included. There are several limitations in current ECMO research, and thus, the retrieval scope should be expanded to conduct in-depth research.

## Data Availability Statement

The original contributions presented in the study are included in the article/supplementary material, further inquiries can be directed to the corresponding author/s.

## Author Contributions

HW designed this study and collected data. SD performed the search. SD and XF rechecked data. HW and SD performed analysis. YL and BY critically revised the work. All authors contributed to the article and approved the submitted version.

## Conflict of Interest

The authors declare that the research was conducted in the absence of any commercial or financial relationships that could be construed as a potential conflict of interest.

## Publisher's Note

All claims expressed in this article are solely those of the authors and do not necessarily represent those of their affiliated organizations, or those of the publisher, the editors and the reviewers. Any product that may be evaluated in this article, or claim that may be made by its manufacturer, is not guaranteed or endorsed by the publisher.
